# Paracrine and Autocrine Effects of VEGF Are Enhanced in Human eMSC Spheroids

**DOI:** 10.3390/ijms232214324

**Published:** 2022-11-18

**Authors:** Irina Kozhukharova, Natalia Minkevich, Larisa Alekseenko, Alisa Domnina, Olga Lyublinskaya

**Affiliations:** Institute of Cytology, Russian Academy of Sciences, 194064 Saint-Petersburg, Russia

**Keywords:** endometrial mesenchymal stem/stromal cells (eMSCs), vascular endothelial growth factor (VEGF), VEGF receptors, spheroids, VEGF knockdown

## Abstract

The mechanisms underlying the therapeutic potential of MSCs are the focus of intense research. We studied human MSCs isolated from desquamated endometrium (eMSCs), which, as previously shown, have high regenerative potential in various disease models. The aim was to evaluate the role of secreted VEGF in stimulating angiogenesis and maintaining eMSC viability and migration, which is important for improving the therapeutic properties of MSCs. We compared three eMSC cultures differing in the level of VEGF secretion: 3D spheroids, monolayer eMSCs, and monolayer eMSCs with VEGF knockdown. Spheroid eMSCs produced higher amounts of VEGF and had the strongest paracrine effect on HUVEC. eMSCs with VEGF knockdown did not stimulate angiogenesis. Monolayered eMSCs expressed VEGFR1, while spheroid eMSCs expressed both VEGFR1 and VEGFR2 receptors. The knockdown of VEGF caused a significant decrease in the viability and migration of eMSCs. eMSCs from 3D spheroids enhanced proliferation and migration in response to exogenous VEGF, in contrast to monolayered eMSCs. Our results suggest that the VEGF–VEGFR1 loop appears to be autocrine-involved in maintaining the viability of eMSCs, and VEGFR2 expression enhances their response to exogenous VEGF, so the angiogenic potential of eMSC can be up- or downregulated by intrinsic VEGF signals.

## 1. Introduction

Mesenchymal stem/stromal cells (MSCs) are being actively studied and are widely used in clinical practice [1]. MSCs are defined as adherent, nonhematologic cells expressing surface markers CD90, CD105, and CD73 and not expressing CD14, CD34, and CD45. They are able to proliferate in vitro for a long time and differentiate into adipocytes, chondrocytes, and osteoblasts when cultured in the relevant medium [2]. Currently, MSCs are isolated from various sources, such as bone marrow, fat, heart, Warton umbilical cord jelly, dental pulp, menstrual blood, and chorion [3]. Initially, it was thought that MSCs could replace damaged tissue during transplantation. However, evidence is now accumulating that the therapeutic effect of MSCs derives from their apparent ability to interact with the cells of the recipient both through direct cell contact [4] and through the secretion of paracrine factors. In the conditioned medium of MSCs, cytokines such as HGF, TGFβ, VEGF, TSG6, PGE2, IL6, MCP-1, and SDF-1 have been found [5,6]. The secreted compounds can influence the repair of damaged tissue by stimulating angiogenesis, inhibiting apoptosis, and regulating the immune response [7,8,9].

Vascular endothelial growth factor VEGFA (hereafter referred to as VEGF) is one of the most important factors that produce angiogenic, trophic, and antiapoptotic effects. Its known roles are the regulation of the proliferation and migration of endothelial cells and the activation of the processes of extracellular proteolysis and vascular formation [9,10]. The production of this growth factor is reduced in adult tissue, with the exception of the female reproductive system, where VEGF production and angiogenesis are continued [11]. VEGF is reexpressed during wound healing and regeneration of damaged tissue, making it a stress-induced protein. VEGF binds to the VEGFR1 and VEGFR2 receptors. Cell signaling occurs through phosphorylation of mitogen-activated protein kinase (MAPK), phosphoinositide 3-kinase and AKT (PI3K/AKT), Src, and Rac [12]. Activation of VEGFR2 determines the processes of migration, proliferation, and differentiation of endothelial cells, the formation of vascular structures, and an increase in cell permeability [13,14]. The functions of VEGFR1 are less well-defined, although it is thought to modulate VEGFR2 signals [14]. There is evidence that VEGFR1 is involved in the differentiation and migration of endothelial cells, but not in their proliferation [15]. It has been shown that the activation of VEGFR1 and VEGFR2, which is necessary for survival and for differentiation of endothelial progenitors, occurs not only in a paracrine but also in an autocrine manner [16] and even in an intracrine manner, i.e., intracellularly [17]. Continuous autocrine expression of VEGF by endothelial cells is required to maintain vascular integrity, cell viability, and protection against autophagy [18]. It has been shown that MSCs isolated from bone marrow, adipose tissue, chorion, and menstrual blood secrete VEGF into the culture medium [19,20,21,22]. Bone marrow MSCs have been shown to promote the survival and differentiation of endothelial progenitor cells through VEGF secretion [23]. Can VEGF secretion in MSCs not only have a paracrine effect but also affect MSCs themselves? This issue remains poorly understood, since a number of studies have not identified the expression of VEGF receptors in MSCs. Expression of VEGFR1 in MSCs from the bone marrow was found in the hypoxic condition, but it significantly decreased during in vitro cultivation [24]. Expression of both VEGF receptors in MSCs was induced during osteogenic and chondrogenic differentiation [25]. It has been shown that differentiation of MSCs in the osteogenic and chondrogenic directions requires endogenous expression of VEGF and activation of its receptors, but this activation is not necessary for adipogenic differentiation [26]. VEGFR1 and VEGFR2 were observed in MSCs upon induction of differentiation into endothelial cells by treatment with high doses of VEGF and hypoxia [27,28]. Upregulation of VEGF and VEGFR2 was detected in endometrial stromal cells during decidualization [29]. This upregulation may contribute to increased angiogenesis and vascular permeability, which is critical for embryo implantation [30]. We have previously shown that using spheroids from endometrial MSCs (eMSCs) is beneficial for the treatment of damaged endometrium and improves fertility [31]. Aiming to investigate the influence of upregulation and downregulation of VEGF and its receptors on the therapeutic potential of eMSCs, this present work compares three types of eMSCs cultures differing in the level of VEGF secretion: 3D spheroids, monolayer eMSCs, and monolayer eMSCs with VEGF knockdown. We evaluated their angiogenic properties, viability, and migration.

## 2. Results

### 2.1. eMSCs Secrete VEGF

**Figure 1:**
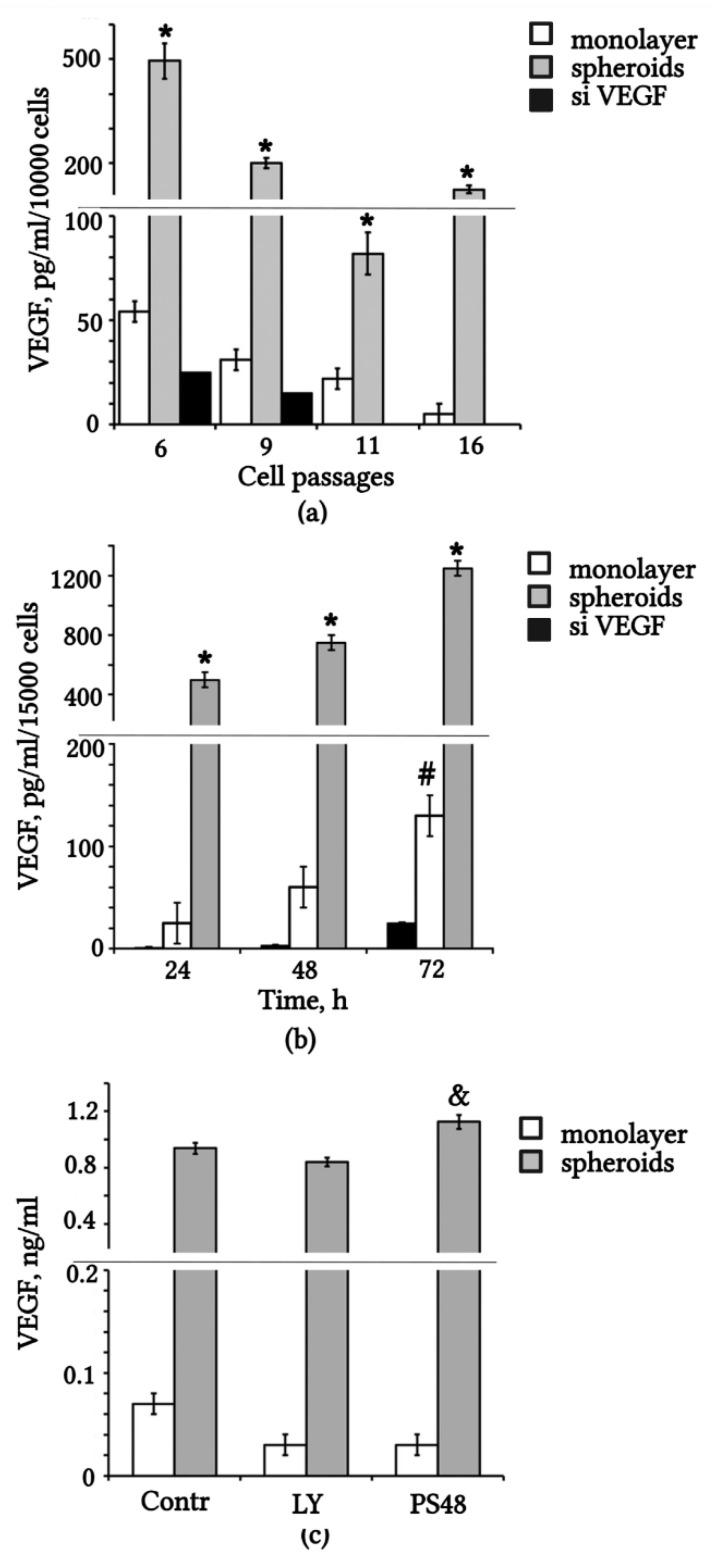
VEGF level is upregulated in conditioned medium from 3D spheroids compared with monolayer eMSCs with/without knockdown of VEGF. (**a**) VEGF concentration in the CM of eMSCs on different passages. (**b**) Dynamics of accumulation of VEGF protein in the CM of eMSCs on passage 6. (**c**) Effect of the inhibition (LY) and activation (PS48) of the PI3K/AKT pathway on VEGF secretion. Monolayer and spheroid eMSCs were treated for 72 h with medium containing 1% FBS (Contr) or supplemented with Ly2090314 (LY) or PS48. VEGF quantity was normalized to volume of the cell medium, and the number of cells in the wells is indicated in the axis caption (in (**a**,**b**)). Values are expressed as means ± SD (n = 3), * *p* < 0.05 spheroids vs. monolayer, # *p* < 0.05 monolayer vs. siVEGF, and & *p* < 0.05 PS48 vs. Contr and LY. CM: conditioned medium; FBS: fetal bovine serum.

The secretion of VEGF by eMSCs was confirmed and quantified using the ELISA technique. We determined the concentration of VEGF in the conditioned medium (CM) of eMSCs depending on expansion in vitro from 6 to 16 passages. The results suggest that VEGF secretion by monolayered eMSCs decreases markedly with increasing passage (Figure 1a). Aggregation of eMSCs into spheroids caused a two- to fivefold increase in the level of VEGF in the CM compared to the same number of monolayer cells (Figure 1a). We transfected eMSCs with siRNA against VEGF and scrambled RNA, and then we compared the concentration of VEGF in CM. Knockdown of VEGF in eMSCs was accompanied by a twofold decrease in VEGF concentration (Figure 1a,b). The level of VEGF consistently increased after 24 h to a maximum value at 72 h in the CM of spheroids and monolayer eMSCs, but not in siVEGF (Figure 1b). All samples of CM contained 1% fetal bovine serum (FBS) to reduce the effect of serum factors on secretion. We evaluated the effect of possible modulators of VEGF expression on its secretion (Figure 1c). Treatment with the small molecule PS48, which is an activator of the PDK1/AKT pathway [32], increased the VEGF content in the CM spheroids (Figure 1c). The Ly2090314 known inhibitor, which blocks the PI3K/AKT signaling pathway, insignificantly reduced the level of VEGF content in the CM-eMSCs (Figure 1c).

### 2.2. Angiogenic Effect of eMSC Conditioned Medium on HUVEC

**Figure 2:**
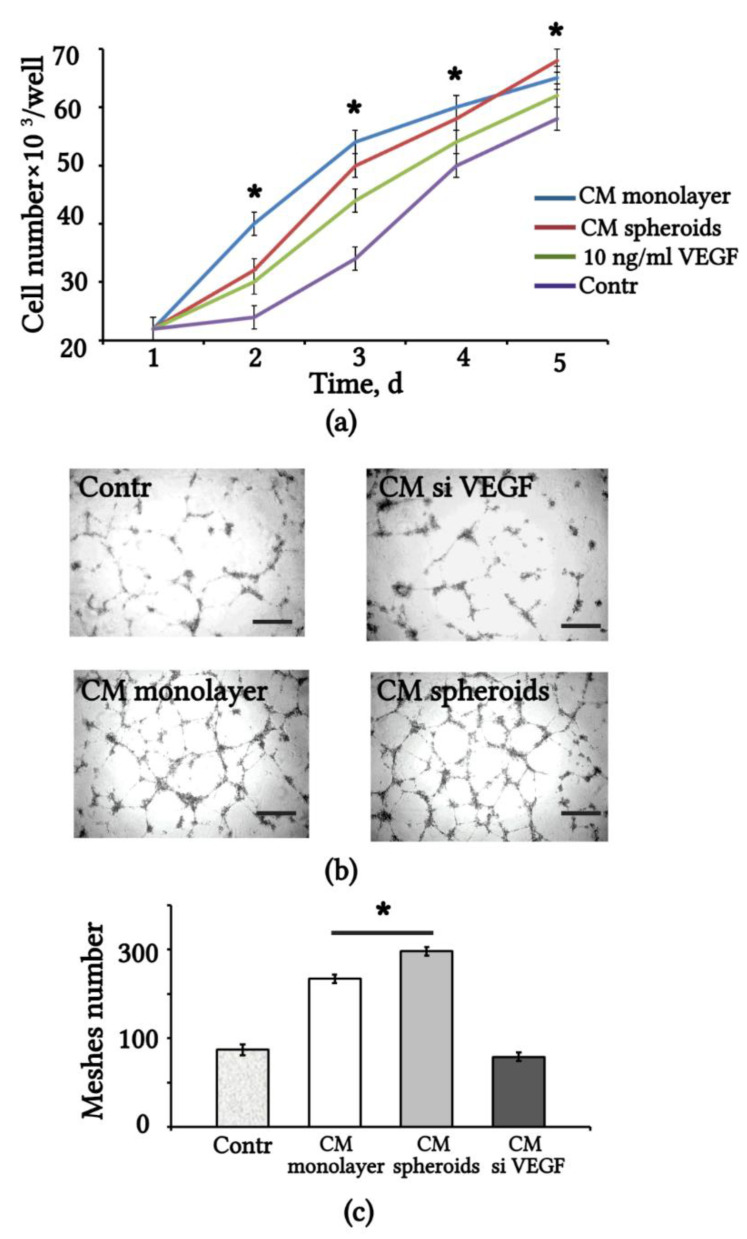
Conditioned medium from 3D spheroids and monolayer eMSCs stimulates proliferation and tube formation of HUVEC. (**a**) HUVEC growth curves when replacing 50% of the growth medium with CM monolayer, CM spheroids, DMEM/F12, added with 1% FBS (Contr) and added with recombinant VEGF. Values are expressed as means ± SD (n = 3), * *p* < 0.05 eMSCs-CM vs. Contr. (**b**) Representative images of HUVEC tube formation on Matrigel taken at 24 h after addition of eMSC-CM. Scale bar 100 µm. (**c**) Quantification of tube formation using the angiogenesis assay measurement tool of software Fiji. Values are expressed as means ± SD (n = 3), * *p* < 0.05 CM spheroids vs. CM monolayer. HUVEC: human umbilical vein endothelial cells; CM: conditioned medium; FBS: fetal bovine serum.

We assessed the angiogenic effect of VEGF released in the CM from monolayer and 3D spheroids of eMSCs. The effect of CM on the proliferation of human umbilical vein endothelial cells (HUVEC) was estimated by counting the number of HUVEC within 5 days of replacing 50% of the basal EGM2 with CM-eMSCs. The HUVEC growth curve is shown in Figure 2a. The results indicate that the CM-eMSCs maintained the proliferation of HUVEC at a higher level than the control growth medium alone. For comparison, we used a culture medium supplemented with recombinant VEGF, and found that CM-eMSCs had the same effect on HUVEC proliferation as the addition of 10 ng/mL VEGF to basal EGM2 (Figure 2a).

We also used the HUVEC tube formation assay to assess the angiogenic activity of CM-eMSCs (Figure 2b). The results show that adding 50% monolayer or spheroid CM caused the increased formation of capillary-like tubular structures; however, CM spheroids stimulated higher mesh formation than the CM monolayer. Treatment with CM-siVEGF induced a significantly weaker effect and did not differ from the control medium (Figure 2c).

### 2.3. Expression of VEGF, VEGFR1, and VEGFR2 in eMSCs

**Figure 3:**
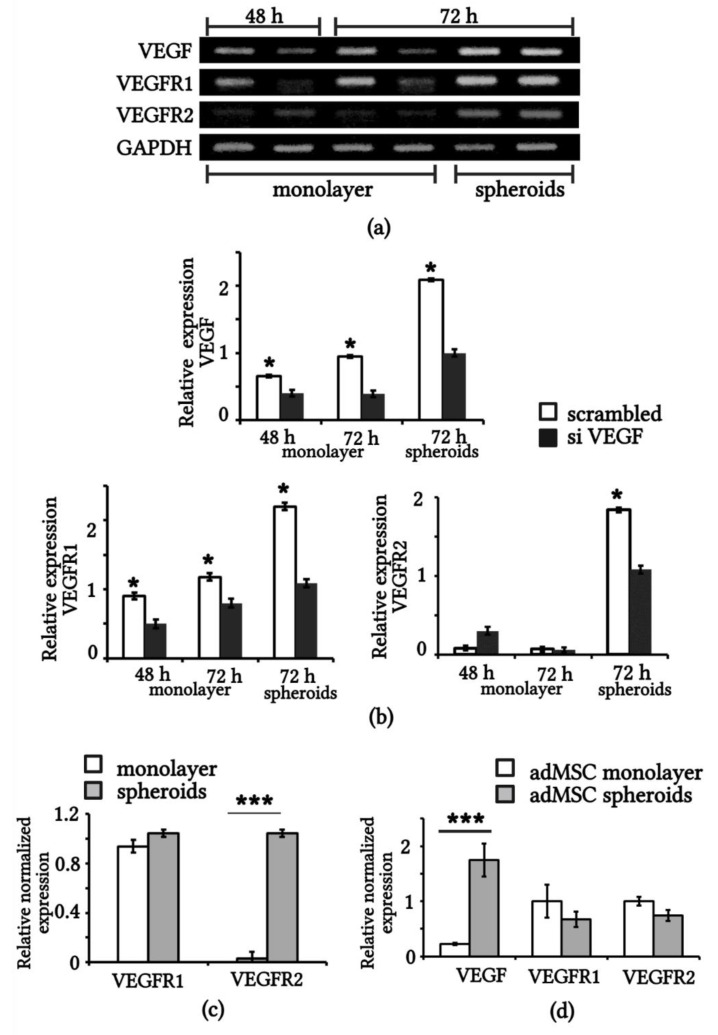
mRNA expression of *VEGF*, *VEGFR1*, *VEGFR2* in MSCs. (**a**) RT-PCR assay of expression of *VEGF*, *VEGFR1*, *VEGFR2* and *GAPDH* genes in eMSCs after transfection with scrambled RNA (odd lines) and VEGF siRNA (even lines) at 48 h and 72 h. (**b**) Quantification of RT-PCR assay normalized to GAPDH (n = 3). (**c**) qRT-PCR assay of *VEGFR1* and *VEGFR2* gene expression in 3D spheroids and monolayer eMSCs. (**d**) qRT-PCR assay of *VEGF*, *VEGFR1* and *VEGFR2* expression in monolayer and 3D spheroid adMSCs. * *p* < 0.05 scrambled vs. siVEGF, *** *p* < 0.01 spheroids vs. monolayer. adMSCs: adipose derived MSCs.

Using RT PCR, we compared the expression of *VEGF, VEGFR1*, and *VEGFR2* genes in eMSCs transiently transfected with short interfering RNAs against *VEGF* (siVEGF) and control RNAs (scrambled; Figure 3a). The densitometry measurements of the VEGF, VEGFR1, and VEGFR2 bands (after normalization with GAPDH) are provided in Figure 3b. Densitometry analysis showed that within 72 h of transfection, *VEGF* gene expression decreased threefold in siVEGF-eMSC compared to the scrambled cells. *VEGF* gene expression was effectively silenced by specific siRNA sequences in monolayered eMSCs and less effectively silenced in spheroid eMSCs (Figure 3b).

All samples of the studied cells had *VEGFR1* gene expression (Figure 3b). *VEGFR2* expression was expectedly low, and there was no obvious difference in the VEGFR2 levels in either the control or siVEGF monolayered eMSCs. However, *VEGFR2* expression was detected in the 3D spheroids of eMSCs (Figure 3b). Using real-time PCR (qRT-PCR), we confirmed the expression of *VEGFR1* in monolayer and spheroid cultures of eMSCs, as well as the induction of *VEGFR2* expression in the spheroid culture (Figure 3c). We decided to look at the expression of *VEGF, VEGFR1*, and *VEGFR2* genes in MSCs from a different source, namely, adipose-derived MSCs (adMSCs). Figure 3d shows the results of the qRT PCR assay using the same primers as for eMSCs. Expression of *VEGF* in spheroid adMSCs was significantly higher than in monolayer adMSCs, but there was no *VEGFR2* induction (Figure 3d).

**Figure 4:**
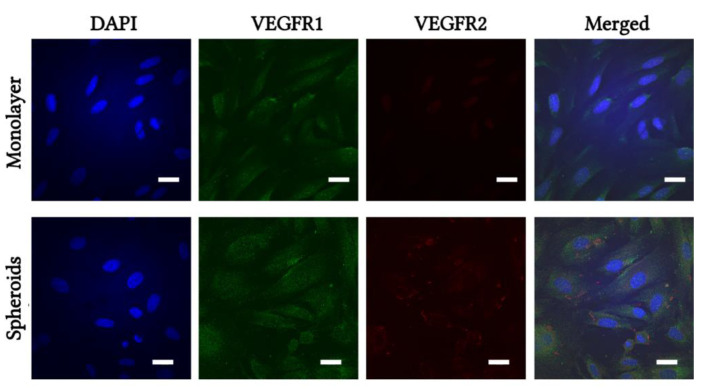
Immunofluorescent staining of VEGFR1 and VEGFR2. Monolayer and 3D spheroids of eMSCs were dissociated and seeded on coverslips, treated with VEGF for 24 h, then fixed and stained with specific antibodies VEGFR1 (green), VEGFR2 (red). Cell nuclei were stained with DAPI (blue). Results show one representative experiment of at least three independent experiments. Scale bar 20 µm.

Expression of VEGFR1 and VEGFR2 proteins was determined by immunofluorescent staining and visualized using confocal microscopy. eMSCs from monolayer and from dissociated spheroids were treated with 10 ng/mL VEGF for 24 h followed by double staining. Figure 4 shows representative images of monolayered and spheroid eMSCs stained with antibodies against human VEGFR1 and VEGFR2. Monolayered and spheroid eMSCs were weakly stained with VEGFR1, but VEGFR2-positive cells were found only in spheroid-derived eMSCs after VEGF treatment (Figure 4).

### 2.4. VEGF Affects the Viability and Proliferation of eMSCs

**Figure 5:**
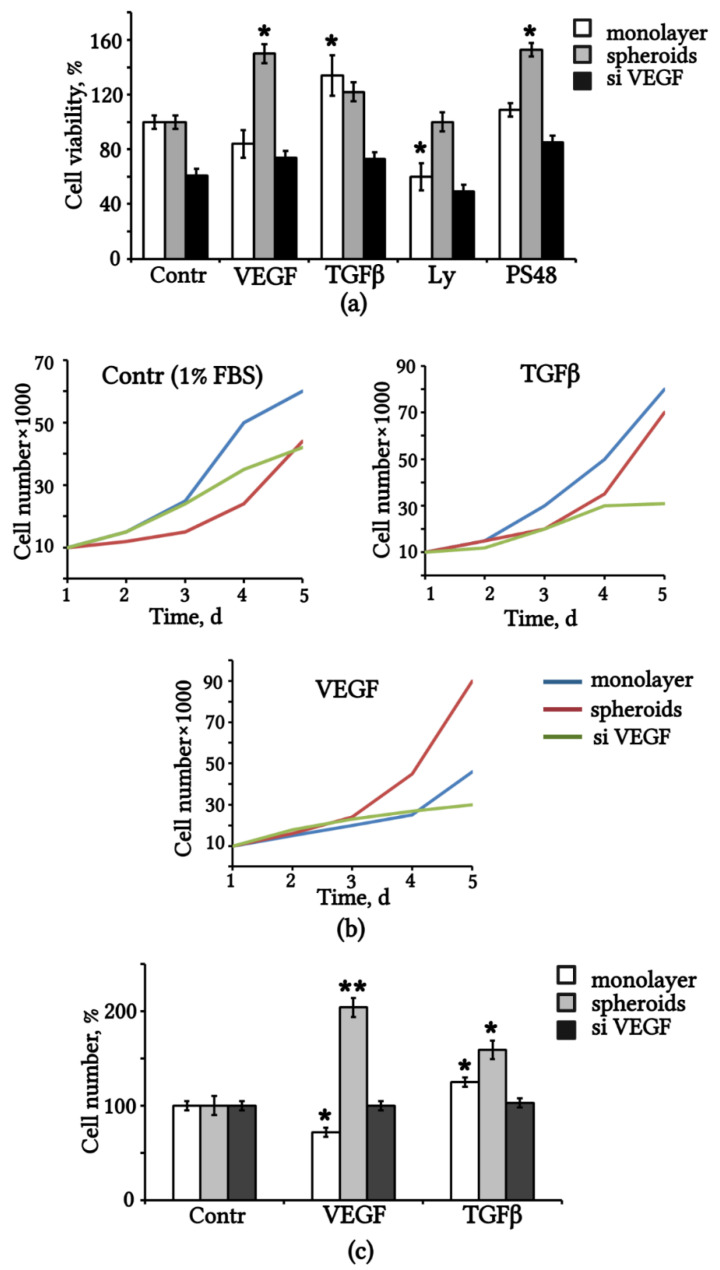
Exogenous and endogenous VEGF affect the viability and proliferation of eMSCs. (**a**) Viability of eMSCs after replacing the medium with 1% FBS (Contr) alone and supplemented with VEGF, TGFβ and small molecules (Ly and PS48) for 48 h, % relative to control monolayer eMSCs. (**b**) Growth curves of eMSCs after dissociation and seeding in the medium with 1% FBS (Contr) alone or supplemented with TGFβ and VEGF respectively. (**c**) Cell number of eMSCs after treatment with VEGF and TGFβ for 5 days, % vs. control medium alone. Results are means  ± SD (n  =  3). * *p* < 0.05; ** *p* < 0.01. FBS: fetal bovine serum.

To determine the influence of exogenous and endogenous VEGF levels on eMSC viability, we compared the survival of eMSCs from monolayer, spheroids, and siVEGF cultures under serum deprivation and treatment with recombinant VEGF or TGFβ using the MTT assay. We used TGFβ as a well-known mitogenic growth factor for MSCs [33]. The results are shown in Figure 5a. VEGF knockdown with siRNA-VEGF significantly reduced eMSC viability compared to the control (scrambled RNA) monolayered eMSCs, and the addition of exogenous VEGF did not restore siVEGF eMSC viability to the control level. The viability of the control monolayer-derived eMSCs in the medium with 1% FBS was high and did not increase with the addition of recombinant VEGF, but the addition of TGFβ significantly promoted viability. Instead, the addition of recombinant VEGF to the medium significantly increased the viability of spheroid-derived eMSCs in contrast to the monolayer culture (Figure 5a). TGFβ treatment also maintained the survival of spheroid eMSCs but was less effective than VEGF. Treatment with small molecules known as inhibitors of the PI3/AKT pathway (LY) and activators of the PDK1/AKT pathway (PS48) confirmed the importance of PI3/AKT pathway activation in maintaining the viability of MSCs. LY reduced the viability of the monolayer eMSCs, and PS48 supported the viability of eMSCs (Figure 5a).

We evaluated the proliferative activity of three eMSC cultures and the effect of exogenous VEGF and TGFβ on the stimulation of cell division. Proliferative activity was determined by counting the number of cells within 5 days of cultivation on a medium with or without the addition of VEGF and TGFβ. Cell growth curves are presented in Figure 5b. The data show that transfection by siRNA-VEGF decreased the proliferation rate in 1% FBS medium, and the addition of VEGF and TGFβ did not rescue it. The influence of the exogenous factors VEGF and TGFβ on the maintenance of eMSCs was assessed by the fold change in the number of cells after the addition of VEGF and TGFβ compared with the control (Figure 5c). The results confirm that exogenous VEGF induces population doubling in spheroid-derived eMSCs but had no such effect on monolayer control and siVEGF eMSCs. Treatment with VEGF even reduced the proliferative activity of monolayer eMSCs compared to the control. TGFβ supported the division of spheroid-derived and monolayered eMSCs.

### 2.5. Migration Activity of eMSCs

**Figure 6:**
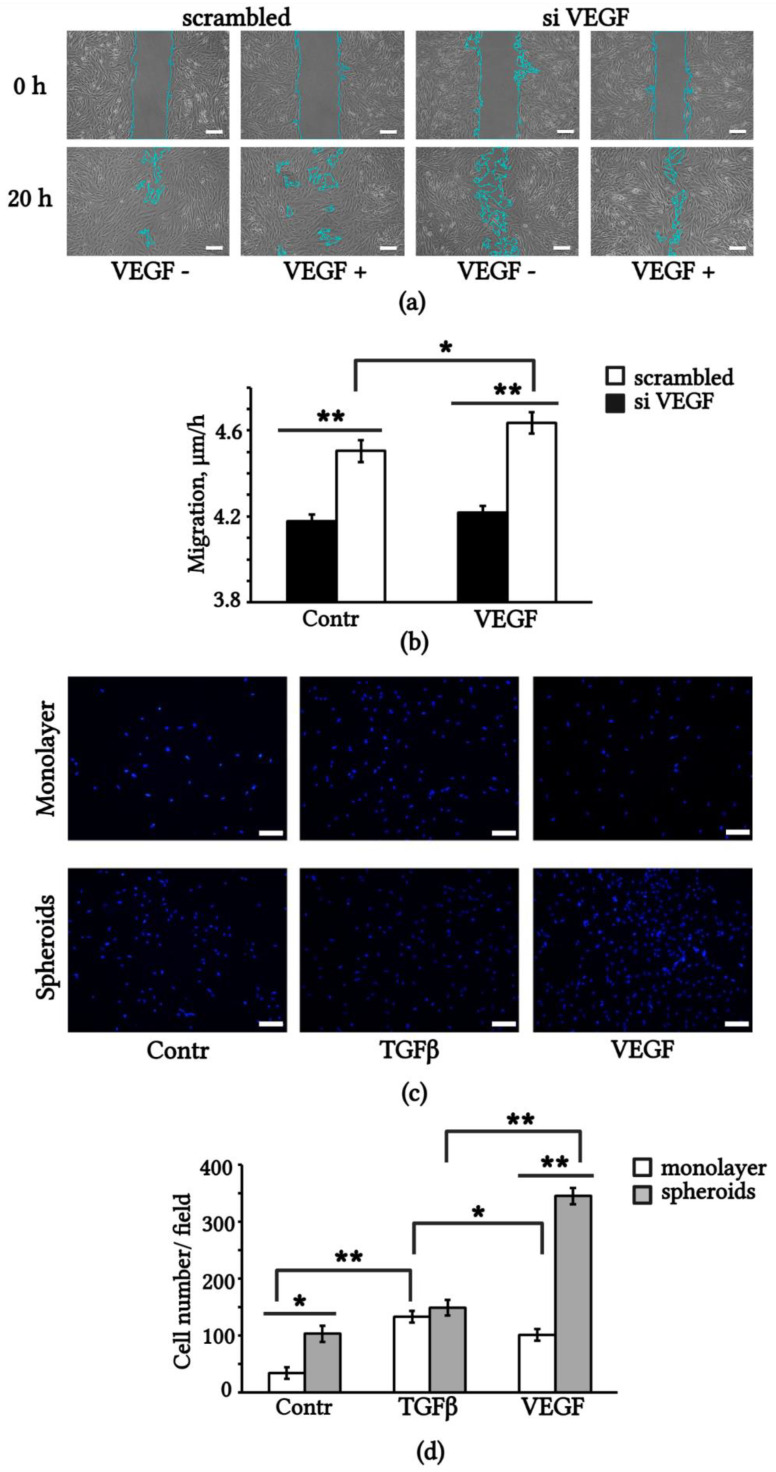
Exogenous and endogenous VEGF affects eMSCs migration. (**a**) Wound healing scratch assay of control monolayer (scrambled) and monolayer VEGF knockdown (siVEGF) eMSCs. The blue lines, generated by the wound healing measurement tool of ImageJ, represent the wound borders. (**b**) Quantification of scratch closure, migration velocity, µm/h. (**c**) Representative images of monolayered and dissociated 3D spheroid of eMSCs stained with DAPI, migrated through transwell inserts to VEGF, TGF and control medium alone; (**d**) Quantification of transwell migration. Scale bar 100 µm. Results are means ± SD (n = 3). * *p* < 0.05, ** *p* < 0.01.

Migration and homing of MSCs to damaged tissue are associated with their therapeutic effects. It has been reported that VEGF can induce the migration activity of MSCs [34]. We assessed the effect of endogenous VEGF expression on the migration activity of eMSCs. We performed scratch wound healing assays with control monolayered eMSCs (transfected with scrambled RNA) and siVEGF (transfected with siRNA against VEGF). The results showed that the migration rate of the control eMSCs was higher than that of the siVEGF-eMSCs (Figure 6a). The addition of 10 ng/mL recombinant VEGF to the medium increased the migration rate of control cells and did not significantly affect the migration rate of siVEGF-eMSCs (Figure 6b). Thus, a knockdown of VEGF expression in siVEGF cells was accompanied by a decrease in the rate of wound healing, and exogenous VEGF did not restore migration to the level of the control cells.

To determine the chemotaxis of eMSCs to exogenous growth factors, we used the method of transwell migration through a polycarbonate membrane with 8 μm pores. We compared monolayered and spheroid-derived eMSCs in terms of mobility in the concentration gradient of VEGF and TGFβ. Representative images of migrated cells staining with DAPI are shown in Figure 6c. Spheroid-derived eMSCs displayed stronger motility in the medium containing 1% FBS (Contr) than monolayered eMSCs. Migration of spheroid-derived eMSCs toward VEGF increased threefold compared to the control medium. Monolayered eMSCs showed a threefold increase in migration to TGFβ (Figure 6d). These data suggest that the aggregation of eMSCs into spheroids enhances their chemotaxis to VEGF, and this property is maintained for at least 5 days after the dissociation of spheroids. It is possible that the expression of VEGFR2 in spheroids, in contrast to the monolayered, provides additional advantages in the mobility of eMSCs towards exogenous VEGF.

## 3. Discussion

In the present work, we have shown that endometrial MSCs (eMSCs), derived from menstrual blood, secrete small amounts of VEGF, but short-term aggregation of eMSCs into spheroids increases VEGF expression and secretion two- to fivefold. Conversely, transfection with siRNAs against VEGF resulted in a threefold knockdown of VEGF expression and secretion. Thus, it is possible to enhance or reduce the angiogenic properties of eMSCs. We tested the functionality of secreted VEGF by assessing its effect on human endothelial cells. Conditioned medium (CM) by eMSCs increased the number of capillary-like structures formed by HUVEC on Matrigel according to the content of VEGF, so CM of spheroids demonstrated a more evaluated paracrine angiogenic effect. eMSCs with VEGF knockdown did not induce HUVEC tube formation. The proliferation of HUVEC was also stimulated by the treatment with CM from eMSCs, similar to the treatment with 10 ng/mL of VEGF, and the differences between the CM of monolayered and spheroid eMSCs were not significant. In our data, the level of VEGF in the CM of spheroids did not exceed 1 ng/mL, while it was less than 100 pg/mL in the CM of monolayer eMSCs. This fact is consistent with the data showing that the CM of MSCs contains a complex of angiogenic factors besides VEGF being involved in maintaining HUVEC proliferation [34]. However, the process of angiogenesis, that is, the formation of new vessels from existing ones, depends to a greater extent on the concentration of VEGF in the eMSCs’ CM.

The results of VEGF protein secretion coincided with the results of VEGF RNA expression: VEGF expression in spheroids was five times higher than in the monolayer. It can be assumed that cell aggregation into spheroids creates conditions for hypoxia, and hypoxia is known to be a strong inducer of VEGF secretion [35]. Previously, we have shown that the expression of the hypoxia-inducible factor HIF-1a is increased in spheroids from eMSCs [36]. These data are consistent with the generally accepted notion that the HIF-1 transcription factor is involved in the regulation of VEGF expression [24]. It should be noted that 3D spheroids of MSCs secrete an increased content of many growth and immunomodulatory cytokines [37]; however, VEGF secretion has always been induced more than other factors in MSC spheroids [38,39]. We hypothesized that eMSCs would not only exhibit paracrine functions due to VEGF secretion but also respond to this secretion due to VEGF receptor expression. Until recently, it was believed that MSCs did not have VEGF receptors on the cell surface. Later studies showed the induction of VEGFR1 expression in BM-MSCs under hypoxia conditions [24] or cocultivation with breast cancer cells [40]. VEGFR1 expression was induced upon BM-MSC differentiation into osteoblasts and endothelium [41,42]. The presence of VEGFR1 on the surface of monocyte-macrophage cells and hematopoietic cells has been noted [43]. Apparently, activation of the VEGFR1 can cause cell migration and differentiation without significantly affecting angiogenesis, even in endothelial cells [44]. VEGFR2 is the main angiogenic marker of endothelial cells: activation of VEGF/VEGFR2 triggers all processes of endothelial growth and survival [45]. The presence of VEGFR2 in BM-MSCs was found only upon induction of endothelial and osteogenic differentiation [28,45].

We find VEGFR2 expression using qRT-PCR assay in spheroid eMSCs and using immunofluorescent staining in dissociated spheroids. Our data show *VEGFR1* expression in all eMSC cells. Its expression increased in proportion to the *VEGF* expression. VEGFR1 and VEGFR2 proteins were detected by immunofluorescence only after stimulation with exogenous VEGF 10 ng/mL. Thus, high levels of VEGF induced the expression of VEGF receptors on the cell membrane. Phosphorylation and activation of VEGFR are topics that should be explored. We tested the effect of exogenous and endogenous VEGF on the survival of the eMSCs themselves. eMSCs with VEGF knockdown showed the lowest viability and proliferative and migratory activity in the low serum medium. Spheroid eMSCs showed a stronger response to the addition of VEGF in the growth medium compared to monolayer eMSCs. Indirect evidence for the involvement of VEGF/VEGFR activation in eMSCs survival may be that there are viability, proliferation, and migration differences between monolayer, spheroid, and siVEGF eMSCs in response to serum starvation and exogenous VEGF stimulation. Analysis of involved signal transduction pathways for the survival and proliferation in eMSCs confirmed the involvement of PI3/AKT in the activation of the VEGF–VEGFR loop, since inhibition of PI3 kinase reduced viability and blocked proliferation in eMSCs with VEGF knockdown. Treatment with exogenous VEGF or with AKT activator PS48, in contrast, increased viability but not proliferation. These results are consistent with reports that VEGFR1 knockdown blocks proliferation and causes premature aging of the endothelium via the PI3/AKT pathway [46].

It is known that MSCs secrete the multifunctional factor TGFβ and express its receptor, which affects the proliferation and differentiation of MSCs [47]. TGFβ/Smad2/3 signaling may be involved in the regulation of *VEGF* gene expression at the transcriptional level [48,49]. We used exogenous TGFβ treatment of eMSCs to distinguish its possible effect on eMSC proliferation from the direct effect of VEGF. Monolayer eMSCs showed a stronger response to TGFβ treatment than to VEGF in proliferation and migration assays. A particular advantage of spheroid eMSCs over monolayer eMSCs is the high mobility of cells in the transwell chamber in the direction of the VEGF gradient. This effect cannot be associated only with the smaller size of cells from spheroids, since they showed less mobility in the direction of TGFβ than monolayered ones. The results obtained require a detailed study of the molecular mechanisms, but it can be assumed that short-term compaction of eMSCs in the spheroids increases plasticity and shifts the differentiation potential of eMSCs. Our results are confirmed in other studies, indicating better survival, mobility, and angiogenic potential of MSCs subjected to aggregation into spheroids [50,51,52]. These studies used adipose MSCs and showed activation of the SDF1–CXCR4 loop in 3D spheroids of adipose MSCs. Our work has shown induction of VEGF expression, but not VEGFR expression in spheroids from adipose-derived MSCs. A feature of our study is the detection of not only a high level of VEGF secretion but also the expression of two VEGF receptors in eMSCs spheroids. This expression determines eMSCs’ increased angiogenic potential and viability in the presence of high doses of VEGF within at least five days of dissociation. This feature may be due to the endometrial origin of eMSCs, since the central role of VEGF and its two receptors in endometrial angiogenesis and vascular permeability, which are critical for preparing the endometrium for embryo implantation, has been experimentally established [30]. We expect to confirm the results on a larger number of donors, since the high variability of MSC properties from different donors is known.

In conclusion, our study showed that short-term aggregation of a monolayer culture of eMSCs into spheroids triggers functional changes in cells, such as increased expression of VEGF and VEGFR1, induction of VEGFR2 expression, and increased cell viability and motility. These changes were accompanied by an increase in the angiogenic potential of eMSCs. The transition from adhesive to spheroid eMSC culture shifted the cellular response to induction by exogenous growth factors: from TGFβ in monolayered eMSCs to VEGF in spheroid eMSC. Knockdown of VEGF gene expression, on the contrary, reduced the viability and migration of eMSCs. Such plasticity of eMSCs holds promise for controlling the balance of secreted growth factors and MSC receptors in the required direction for therapeutic purposes.

## 4. Materials and Methods

### 4.1. Cells

Human endometrial mesenchymal stem/stromal cells (eMSCs) were obtained from the shared research facility Vertebrate Cell Culture Collection of the Institute of Cytology, RAS. These cells were derived from a desquamated endometrium of menstrual blood from a healthy 27-year-old woman, as has previously been described [53]. eMSCs were cultured in DMEM/F12 growth medium (Gibco, Grand Island, NY, USA) supplemented with 10% fetal bovine serum (FBS, HyClone, Pasching, Austria), 1% Glutamax (Gibco, Grand Island, NY, USA), and 1% penicillin–streptomycin (Gibco, Grand Island, NY, USA) at 37 °C in a humidified chamber with 5% CO_2_. Cells were subcultured twice weekly with 0.05% EDTA–trypsin solution (Gibco) for 6–12 passages. eMSCs express CD105, CD44, CD73, CD90, CD146, and CD29 surface markers and are negative for the hematopoietic markers CD34 and CD45. Endometrial MSCs possess the ability to differentiate into adipocytes, chondrocytes, and osteoblasts [31]. In the experiments, we compared three eMSC cultures: control monolayered, short-term aggregated into 3D spheroids, and monolayered eMSCs transfected with short interfering RNAs against VEGF. All cells were seeded in 96-well plates in DMEM/F12 growth medium containing 1% serum.

Human adipose-derived mesenchymal stem/stromal cells (FMSCs) were obtained from the Almazov National Medical Research Center of the Ministry of Health of Russia. The cultivation conditions were the same as for the eMSCs.

Human umbilical vein endothelial cells (HUVEC) were obtained from the Almazov National Medical Research Center of the Ministry of Health of Russia. Cells were cultured in EGM2 medium (PromoCell, Heidelberg, Germany) supplemented with 2% FBS (PromoCell, Germany), 1% penicillin–streptomycin (Gibco), and hEGF 5 ng/mL, hydrocortisone-100 0.2 µg/mL, hVEGF 0.5 ng/mL, hbFGF 10 ng/mL, R3 IGF 22 ng/mL, ascorbic acid-500 1 µg/mL, and heparin 22.5 ng/mL (Supplement Pack, PromoCell, Germany). Cells were subcultured once weekly with Accutase (Gibco, Grand Island, NY, USA) up to passage 6.

### 4.2. Spheroid Formation

Spheroids were formed from eMSC using nonadhesive round-bottomed 96-well plates (BioFil, Guangzhou, China) coated with 2-hydroxyethyl methacrylate (HEMA; Sigma-Aldrich, St. Louis, MO, USA). To do this, we placed 15,000 cells in each well of a plate in 35 µL of growth medium. Cells spontaneously aggregated into spheroids for 48 h, and then a serum-free medium was added so that the final serum content in the medium was 1%, and it was cultured for 24–72 h.

### 4.3. Conditioned Medium Collection

To analyze the monolayered cells, we seeded eMSCs with a density of 15,000 cells per well on flat-bottom 96-well plates (Nunc, Roskilde, Denmark). The next day, we washed cells with PBS and added 0.2 mL DMEM/F12 with 1% FBS. To obtain conditioned medium (CM) from 3D spheroids, we seeded the same number of eMSCs on round-bottomed 96-well plates, and after spheroid formation for 48 h, washed with DMEM/F12 and added 0.2 mL DMEM/F12 with 1% FBS. Cells were incubated in standard conditions (5% CO_2_; 37 °C) for 24, 48, and 72 h. Then, CM was collected, centrifuged at 400× *g* for five minutes to remove debris, and stored at −20 °C prior to assay. The supernatants were used for ELISA and angiogenic assays, and cells were used for RNA isolation.

### 4.4. Enzyme-Linked Immunosorbent Assay

The protein concentration of VEGF in the CM was measured using the ELISA kit DuoSet Human VEGF (R&D-system, Minneapolis, MN, USA) following the manufacturer’s instructions. Quantification was performed according to the standard curve of recombinant human VEGF.

### 4.5. MTT Test

MTT colorimetric assay was used to assess cell viability. To compare three types of eMSCs—monolayer (control), spheroids, and monolayer siVEGF—we dissociated all cells with Accutase (Gibco, Grand Island, NY, USA). Then, we seeded 5000 cells/well in 100 μL of growth medium into 96-well tissue culture plates, which were incubated overnight at 37 °C in a humidified chamber with 5% CO_2_. The next day, the growth media were changed for 1% FBS, and 24 h later, we added tested factors or vehicles. Cells were treated with the following reagents: (1) recombinant human endothelial growth factor (VEGF) 10 ng/mL (Gibco, Grand Island, NY, USA); (2) recombinant human transforming growth factor beta1 (TGFβ) 0.1 ng/mL (Invitrogen, Waltham, MA, USA); (3) LY294002 (LY), a specific inhibitor of phosphatidylinositol-3-kinases, 25 µM (Abcam Biochemicals, Cambridge, UK); (4) PS48 allosteric activator of the PDK1/AKT signaling pathway 10 µM (Abcam Biochemicals, Cambridge, UK). After an incubation period of 48 h, MTT [3-(4,5-dimethylthiazolyl-2)-2,5 diphenyltetrazolium bromide] solution (Sigma-Aldrich, St. Louis, MO, USA) was added. The absorbance of the samples was measured at 570 nm using a microplate reader (Varioskan LUX, Thermo Fisher Scientific Inc., Waltham, MA, USA). The cell viability was calculated as follows: Absorbance of tested samples/Absorbance of control × 100%.

### 4.6. siRNA Transfection

To silence the expression of VEGF, we transiently transfected eMSCs with specific siRNAs consisting of a mixture of three target double-stranded RNA molecules. VEGF short interfering RNA (siVEGF) and scrambled control siRNA were purchased from Santa Cruz Biotechnology, Dallas, TX, (USA). 250 × 103 eMSCs per 1 mL of culture medium without antibiotics were seeded in 6-well plates. The next day, siRNA transfection medium (Santa Cruz Biotechnology, Dallas, TX, USA) was applied to dilute the siRNA and lipofectamine RNAiMAX (Invitrogen, Waltham, MA, USA). siRNA was diluted to a concentration of 37.5 ng/mL and mixed with lipofectamine to form a transfection complex, then 200 μL of the mixture was added to 800 μL of culture medium to obtain a final concentration of 5 nM siRNA after addition to cells. First, cells with transfection complexes were incubated for 6 h, then serum and antibiotics were added and incubated for 24 h, and lastly the culture medium was completely changed. Gene silencing was verified by RT-PCR.

### 4.7. In Vitro Wound Healing Assay

Two-well silicone inserts (Ibidi, Graefelfing, Germany) in 24-well plates were used to create a defined cell-free gap. We seeded 25,000 cells in a growth medium on both sides of the insert and incubated them overnight until a confluent monolayer had formed. Then, the inserts were removed, the cells were washed with PBS, and 1 mL of fresh growth medium (with or without VEGF) was added to the wells. Phase-contrast eMSC images were taken at 0, 8, and 24 h to monitor gap closure. The gap size was measured as a function of time using the Fiji/ImageJ plugin <Wound-Healing-Tool> (https://github.com/MontpellierRessourcesImagerie/imagej_macros_and_scripts/wiki/Wound-Healing-Tool), and the migration rate was calculated using Microsoft Excel.

### 4.8. Transwell Migration Assay

Transwell chambers with 8 µm size pores for 24-well plates (Thermo Fisher Scientific, Roskilde, Denmark) were used to monitor eMSC motility to VEGF and TGFβ gradient concentration. The eMSC cultures (monolayer and spheroids) were dissociated with Accutase (Gibco) and washed with PBS; then, 55,000 cells in 100 µL of growth medium with 1% FBS were applied to the upper chamber. Next, 500 μL the same medium (control) or supplied with recombinant factors (10 ng/mL VEGF or 0.1 ng/mL TGFβ) was added to the lower chamber. After incubation for 24 h at 37 °C, non-migrated cells on the upper surface of the insert were removed with a cotton swab, and the cells migrated through pores were fixed in 4% paraformaldehyde (Sigma-Aldrich, St. Louis, MO, USA) for 20 min and stained with DAPI (4′,6-diamidino-2-phenylindole, dihydrochloride, Darmstadt, Germany) at a concentration of 1 μg/mL. Images of the migrating cells were taken using 10 × objectives on a fluorescence microscope (Acsioscope Zeiss, Jena, Germany).

### 4.9. RT-PCR and qRT-PCR Assays

To analyze gene expression, we isolated total RNA with Aurum™ Total RNA Mini Kit (BioRad, Hercules, CA, USA) according to the manufacturer’s instructions. RNA was quantified in the NanoDrop ND-1000 Spectrophotometer (NanoDrop Technologies, Inc., Wilmington, DE, USA). cDNA was synthesized from total RNA using the Revert Aid H Minus First-Strand cDNA Synthesis Kit (Fermentas, Vilnius, Lithuania). It was subsequently amplified with specific primers using DreamTaq™ PCR Master Mix 2X (Thermo Fisher Scientific, Waltham, MA, USA). The electrophoresis of amplified products was performed in 2% agarose gel with TAE buffer and ethidium bromide. DNA ladder 100 kb (Fermentas, Vilnius, Lithuania) was used for molecular weight markers. Amplified products were visualized in UV light (302 nm) with a Transilluminator and registered with a digital Canon camera (Canon, Tokyo, Japan). Quantification was carried out using densitometry analysis in the Scion Image program. For qRT-PCR, cDNA was amplified with specific primers, using EvaGreen^®^ dye (Biotium, Fremont, CA, USA) and DreamTaq™ PCR Master Mix 2X (Thermo Fisher Scientific, Waltham, MA, USA) in the Bio-Rad CFX-Opus 96 real time system (Bio-Rad, Singapore), according to the kit’s enclosed protocol. Expression of target genes was normalized to the GAPDH gene. Primers were designed using the Primer-Blast NCBI program. Primer sequences were as follows:

VEGF:
F: 5′-CTACCTCCACCATGCCAAGT-3′
R: 5′-GCAGTAGCTGCGCTGATAGA-3′
product size 81 bp
VEGFR1:
F: 5′-CGACCTTGGTTGTGGCTGACT-3′
R: 5′-CGGTTCTGGTTGGTGGCTTTG-3′
product size 82 bp
VEGFR2:
F: 5′-AACAAAGTCGGGAGAGGA-3′
R: 5′-TGACAAGAAGTAGCCAGA-3′
product size 87 bp
GAPDH
F: 5′-GAGGTCAATGAAGGGGTCAT-3′
R:5′-AGTCAACGGATTTGGTCGTA-3′
product size 100 bp

All amplifications were performed in triplicates. Experiments were repeated at least three times.

### 4.10. Tube Formation Assay

First, 96-well plates were treated with a cold growth factor reduced Matrigel (BD Biosciences, San Jose, CA, USA) solution in a volume of 50 µL and incubated for 30 min for gelation. HUVEC were placed on precoated wells (3 × 104 cells/well) in 50 μL of EGM basal medium. Then, 50 μL of tested CM or control DMEM/F12 + 1% FBS was added to the cells and incubated for 20 h at 37 °C in a humidified chamber with 5% CO_2_. Images at 10× magnification were obtained using a phase contrast inverted microscope (Nikon eclipse TS100, Tokio, Japan) and Canon EOS1000, Tokio, Japan. The angiogenic properties of the conditioned medium were quantified by the total length of the tubes and the number of meshes using the Fiji-ImageJ software with an angiogenesis analyzer extension.

### 4.11. Immunofluorescence

eMSCs from monolayer or spheroids were dissociated with Accutase and seeded on coverslips in 12-well plates. After cell attachment, the culture medium was changed to fresh with 1% FBS, and cells were incubated for 24 h at 37 °C, 5% CO_2_. Then, 10 ng/mL of recombinant VEGF or vehicle was added for 48 h. Cells on coverslips were washed with PBS, fixed with 4% paraformaldehyde (Sigma-Aldrich, St. Louis, MO, USA) in PBS for 20 min, and permeabilized with 0.1% Triton X-100 (Merck, Darmstadt, Germany) for 15 min. Aspecific binding was blocked by incubation with 2% goat serum and 1% BSA (all Sigma Aldrich, St. Louis, MO, USA) in PBS for 60 min at room temperature. The primary antibodies used were rabbit polyclonal to human FLT1 (VEGFR1 720043; Thermo Fisher Scientific, Waltham, MA, USA) and mouse monoclonal to human KDR/VEGFR2 (CD309 conjugated PE, BD Pharmingen). Anti-VEGFR1 antibodies were dissolved 1:100 in a blocking buffer and incubated overnight at 4 °C. The next day, secondary antibodies goat anti-rabbit Alexa 488 and VEGFR2-conjugated PE were applied for 1 h at room temperature. The nuclei were stained with DAPI (4′,6-diamidino-2-phenylindole, dihydrochloride, Merck, Darmstadt, Germany) at a concentration of 1 μg/mL. Then, the coverslips were mounted in ProLong Gold antifade reagent (Thermo Fisher Scientific, Waltham, MA, USA) and imaged with confocal laser scanning microscopy (Olympus FV3000, Olympus Corporation). Incubation with secondary antibodies alone was used as a negative control.

## Data Availability

The data that support the findings of this study are available from the corresponding author upon reasonable request.
